# Investigating the Effectiveness of Low-Level Laser in Reducing Root Resorption of the Upper Incisors During Intrusion Movement Using Mini-Implants in Adult Patients With Deep Overbite: A Randomized Controlled Clinical Trial

**DOI:** 10.7759/cureus.35381

**Published:** 2023-02-23

**Authors:** Amer R. Nasser, Kinda Sultan, Mohammad Y Hajeer, Omar Hamadah

**Affiliations:** 1 Department of Orthodontics, University of Damascus Faculty of Dentistry, Damascus, SYR; 2 Department of Oral Medicine, University of Damascus Faculty of Dentistry, Damascus, SYR

**Keywords:** adult patients, upper incisor intrusion, volumetric analysis, cone beam computed tomography – cbct, overbite, intrusion, mini-implants, root resorption, low-level laser, deep bite

## Abstract

Background: Deep bite is a common characteristic of malocclusion, and many methods are used to treat it, including mini-implants used for the intrusion of the upper incisors. Orthodontically induced inflammatory root resorption (OIIRR) is an inevitable and unexpected side effect of orthodontic therapy. However, resorption of the root could be affected by the type of tooth movement, such as intrusion. Several studies have indicated the effectiveness of low-level laser therapy (LLLT) in accelerating orthodontic movement, but studies that have evaluated the role of this laser in reducing the risk of OIIRR have been limited. This trial aimed to investigate the effectiveness of LLLT in reducing the resorption of the roots of the upper incisors during their intrusion in the context of deep bite correction.

Materials and methods: Thirty patients (13 males, 17 females; mean age 22.4±3.37 years) with deep overbite were recruited and allocated to the laser or the control groups. Mini-implants were inserted between the roots of the upper central incisors and the lateral incisors from the labial aspect at the gingival-mucosal junction on both sides with a force of 40 g on each side through an NiTi coil spring. A low-level laser (Ga-Al-As) with 808 nm wavelength in a continuous mode, with the parameters 250 milliwatt power output, 4 Joules/point energy density, and 16 seconds irradiation per point, was applied to the root of each of the upper incisors. The laser was applied on the first day of the upper incisor intrusion (T1), then on days 3, 7, and 14 of the first month. In the second month, the laser was applied every 15 days, adjusting the spring strength every four weeks until the end of the intrusion stage (T2), which was determined by reaching a normal overbite. As for patients in the control group, the strength of the nickel-titanium springs was adjusted every four weeks to the required strength of 40 g on each end until reaching a normal overbite.

Results: There was a volumetric decrease in both groups' upper central and lateral incisors roots, and this decrease was statistically significant (P<0.001). However, the difference between the two groups was not statistically significant in each central and lateral incisor volume root (P=0.345 and 0.263 for U1 and U2, respectively). Also, both groups had a linear decrease in upper central and lateral incisors roots, which was statistically significant (P<0.001). At the same time, the difference between the two groups was not statistically significant in each central and lateral incisor root length (P=0.343 and 0.461 for U1 and U2, respectively).

Conclusion: The low-level laser irradiation using the current protocol did not significantly affect the amount of root resorption induced by incisor intrusion in the experimental group compared to the control group.

## Introduction

Deep bite is a common characteristic of malocclusion, which appears when the mandibular incisor crowns overlap excessively vertically with the maxillary incisors when the teeth are in centric occlusion [1]. As its treatment and the choice of correction method depend mainly on the diagnostic data and its etiology, among the therapeutic options are the intrusion of anterior teeth, extrusion of posterior teeth, or both [2-4].

 A common method of incisor intrusion used involves appliances such as utility arches, three-piece intrusion arches, or reverse-curved arches, as well as the use of mini-implants. The first report on using mini-implants for this purpose was given by Creekmore and Eklund in 1983 [5]. Even though mini-implants are more invasive, they have advantages such as immediate loading and multiple placement sites, eliminating undesirable side effects such as protrusion. However, with a light amount of force and any method of intrusion, some degree of root resorption is always expected [6].

Orthodontically induced inflammatory root resorption (OIIRR) is an inevitable and unexpected side effect of orthodontic therapy [7]. However, root resorption could be affected by the type of tooth movement [8]. In addition, the long duration of orthodontic treatment leads to an increased risk of root resorption, gingival infections, and dental caries, and this has led to an increase in the demand to find the best way to increase tooth movement with the least possible amount of these complications [9].

In recent years, low-level laser therapy (LLLT) has been used to improve the biomechanics of orthodontic movement. The results have shown an increase in the rate of dental movement, whether it is the acceleration of canine retraction or accelerating the intrusion of the posterior teeth [10,11], with a decrease in the risk of root resorption and an adequate biological response in the periodontal ligament [12] in addition to its use in reducing dental pain [13].

Several studies have indicated the effectiveness of the LLLT in accelerating orthodontic movement, but studies that have evaluated the role of this laser in reducing the risk of OIIRR have been limited [7]. Moreover, the effects of laser in reducing root resorption have been studied with orthodontic movements such as en masse anterior retraction, canine retraction, and tilting of the premolars before extraction, but they have not been studied with the intrusion movement [7,14,15]. Therefore, this study aimed to investigate the effectiveness of low-level lasers in reducing the resorption of the roots of the upper incisors during their intrusion in the context of deep bite treatment.

## Materials and methods

Study design and settings

This study is a two-arm, parallel-group, randomized controlled trial (RCT) investigating the effectiveness of low-level laser in reducing the resorption of the roots of the upper incisors. The CONSORT declaration served as a guide for optimally writing the article. The study was carried out at the Department of Orthodontics and the Research Unit for Lasers at Damascus University Faculty of Dentistry between November 2019 and July 2022. Ethical approval was obtained from the Local Research Ethics Committee at the University of Damascus (UDDS-731-02112019/SRC-2246). This RCT is registered in the Clinical Trials database (ID: NCT05734092) and was funded by the University of Damascus Postgraduate Research Budget (Ref no: 891262195DEN)

Sample size calculation

The sample size was calculated using the Minitab® Version 18 (Minitab Inc., State College, Pennsylvania, USA) program according to a significant mean difference of 0.54 mm of root resorption with a standard deviation of 0.47 mm for the same variable [16], the statistical power of 80%, and a significance level of 0.05; a two-sample t-test was used, and the minimum sample size needed for each group was 13. This number was increased to 15 per group to compensate for a 10% possible dropout.

Patient recruitment and entry in this trial

**Figure 1:**
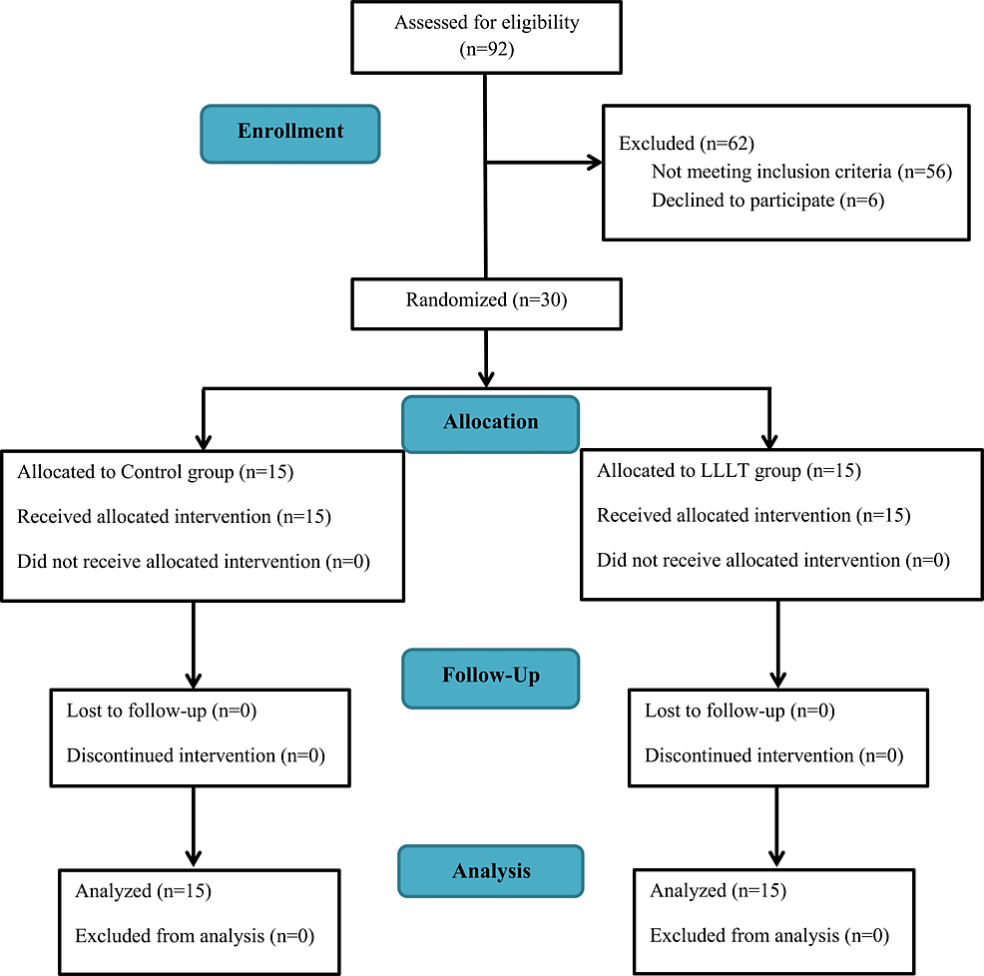
CONSORT flow diagram of patients' recruitment, follow-up, and entry into data analysis CONSORT: Consolidated Standards of Reporting Trials; LLLT: low-level laser therapy.

Participants were chosen from patients who visited Damascus University's Department of Orthodontics and Dentofacial Orthopedics. Clinical examination was conducted on 92 patients. The patient was deemed eligible for the study if the patient satisfied the following inclusion requirements: (a) aged between 18 and 30 years, (b) overbite >4 mm, (c) Class I or II molar and canine relationship malocclusion, (d) maxillary anterior crowding <2 mm, (e) maxillary incisors positioned below the functional occlusal plane, (f) incisor display of 2-3 mm or more at rest, and (g) the facial growth pattern is normal or horizontal. Patients were excluded if (a) the maxillary incisors had a history of any trauma or endodontic treatment, (b) there was an increased inclination of the maxillary incisors toward the labial, (c) they had any systemic disease, or (d) they exhibited poor oral hygiene. The Consolidated Standards of Reporting Trials (CONSORT) flow diagram of patients' recruitment, follow-up, and entry into data analysis is given in Figure 1.

Randomization and blinding

Patients were divided into two groups: the laser group and the control group, with an allocation ratio of 1:1 using a simple randomization technique. Each patient was instructed to choose a folded piece of paper from a box containing 30 pieces, 15 of which were marked with the word "laser" and 15 marked with the term "control." The patient was put into one of the two groups based on their chosen piece. The development of the random allocation sequence, the enrolment of participants, and the assignment to the intervention were all delegated to a member of the academic staff who was not involved in the study activity. The primary researcher or patients could not be rendered blind. Therefore, only outcome assessments were blinded.

Clinical procedures

**Figure 2:**
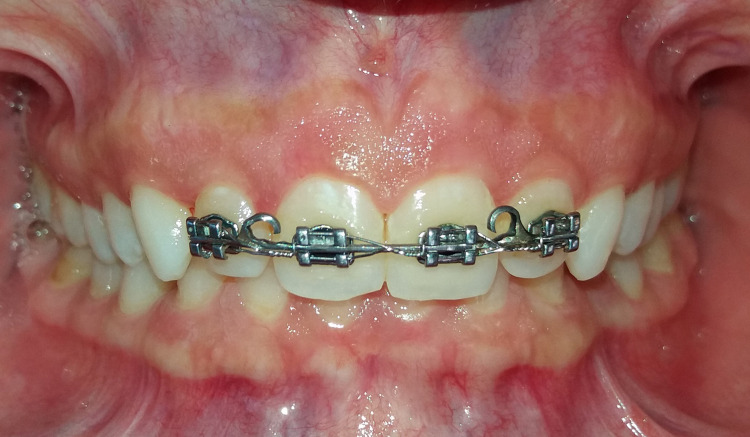
The soldered hooks were placed between the central and lateral incisors on each side.

After completing the diagnostic procedures, the fixed orthodontic appliances of the MBT prescription and 0.022-inch slot height (Master Series®, American Orthodontics, Sheboygan, Wisconsin, USA) were attached to the upper incisors of both groups, where the segmented adhesive technique was used [17]. In the beginning, round Nitinol (NiTi) wires with diameters of 0.012 inches, 0.014 inches, and 0.016 inches were used in sequence. Then, 0.016×0.022 inch, 0.017×0.025 inch, and 0.019×0.025 inch NiTi archwires were used in the following stage. Archwire replacement was done every three weeks until reaching the stainless-steel rectangular wire with a diameter of 0.019x0.025 inch. Soldered hooks were placed between the central and lateral incisors on each side (Figure 2).

**Figure 3:**
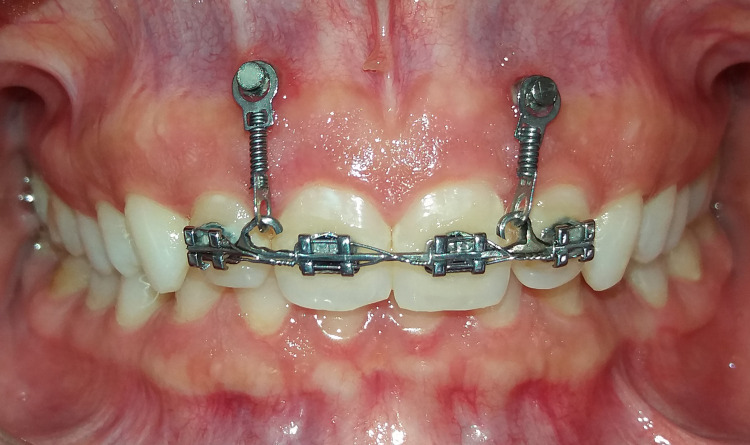
Coil springs were extended between the mini-implants' heads and the soldered hooks.

After completing the leveling and alignment stage, self-drilling mini-implants with a length of 8 mm and a diameter of 1.4 mm HUBIT® Standard Mini Screw (HUBIT, Gyeonggi-do, Korea) were inserted between the roots of the upper central incisors and the lateral incisors from the labial aspect at the gingival-mucosal junction on both sides [18]. This was done within the distance of safe placement of mini-implants, according to a study by Yang et al. [19]. The loading of these mini-implants was accomplished immediately following insertion with a force of 40 g, according to the study of Aras and Tuncer and Burstone [20,21], on each side through an NiTi locking spring extending from the head of the mini-implants to the hooks welded on the archwire (Figure 3). The amount of force was then verified using a force gauge.

Laser application and dosimetry (LLLT group)

**Figure 4:**
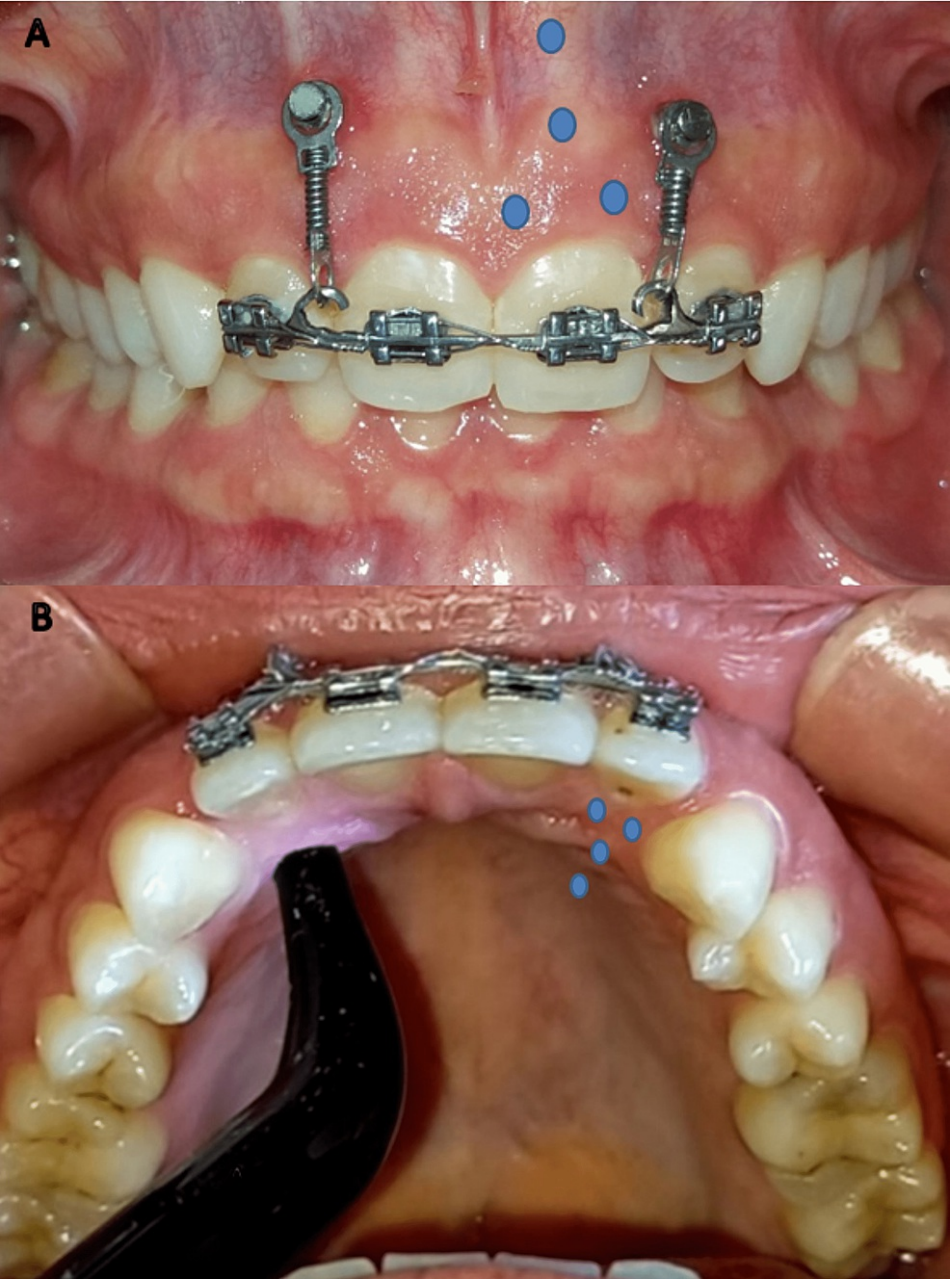
Laser irradiation in the experimental group. A: The blue dots indicate the areas of irradiation on the vestibular surface of the tooth. B: The blue dots indicate the areas of irradiation on the palatal surface of the tooth.

In the experimental group (LLLT group), a semiconductor gallium-aluminum-arsenide (Ga-Al-As) diode laser (Klas-DX6182, Konftec Corporation, New Taipei City, Taiwan) was used with 808 nm wavelength in a continuous mode, with the following parameters: 250 milliwatt power output, 4 Joules/point energy density, and 16 seconds irradiation per point [10]. The laser was applied to the root of each of the upper incisors at eight points (four points from the labial side and four points from the palatal side) with a total application time of 128 seconds for each tooth. The irradiation head (tip) was placed from the labial side of the root in the center of each of the apical and middle thirds and the mesial and distal of the cervical third in contact with oral mucosa (Figure 4). The irradiation head was oriented perpendicular to the root axis [7]. The laser was applied on the first day of the upper incisor intrusion (T1), then on days 3, 7, and 14 of the first month. In the second month, the laser was applied every 15 days, adjusting the spring strength every four weeks until the end of the intrusion stage (T2), which was determined by reaching a normal overbite.

The control group

As for patients in the control group, the strength of the nickel-titanium springs was adjusted every four weeks to the required strength of 40 g on each end until reaching a normal overbite.

Outcome measures: Volumetric and linear assessment of root resorption

Image Acquisition

Cone-beam computed tomography (CBCT) images were taken before the beginning of the intrusion (T1) and at the end of the intrusion (T2). CBCT image was limited to the maxillary jaw only in both groups using a CBCT imaging apparatus (Pax-i3D Green, Vatech, Seoul, Korea) with the following parameters: a field of view of 15 × 15 cm, a voxel size of 0.2 × 0.2 mm, a voltage of 98 kVp, an amperage of 11.2 mA, and 9 seconds scanning time. A horizontal plane parallel to the floor, a consistent head posture, teeth in their maximum intercuspation, and a standard methodology were used to scan every patient. The obtained data were viewed by Ez3D Plus® CDViewer software (Ver. 1.2.6.23, Vatech & Ewoo, Gyeonggi-do, South Korea) and then exported as Digital Imaging and Communication in Medicine (DICOM) files.

Volumetric Assessment of the Roots

**Figure 5:**
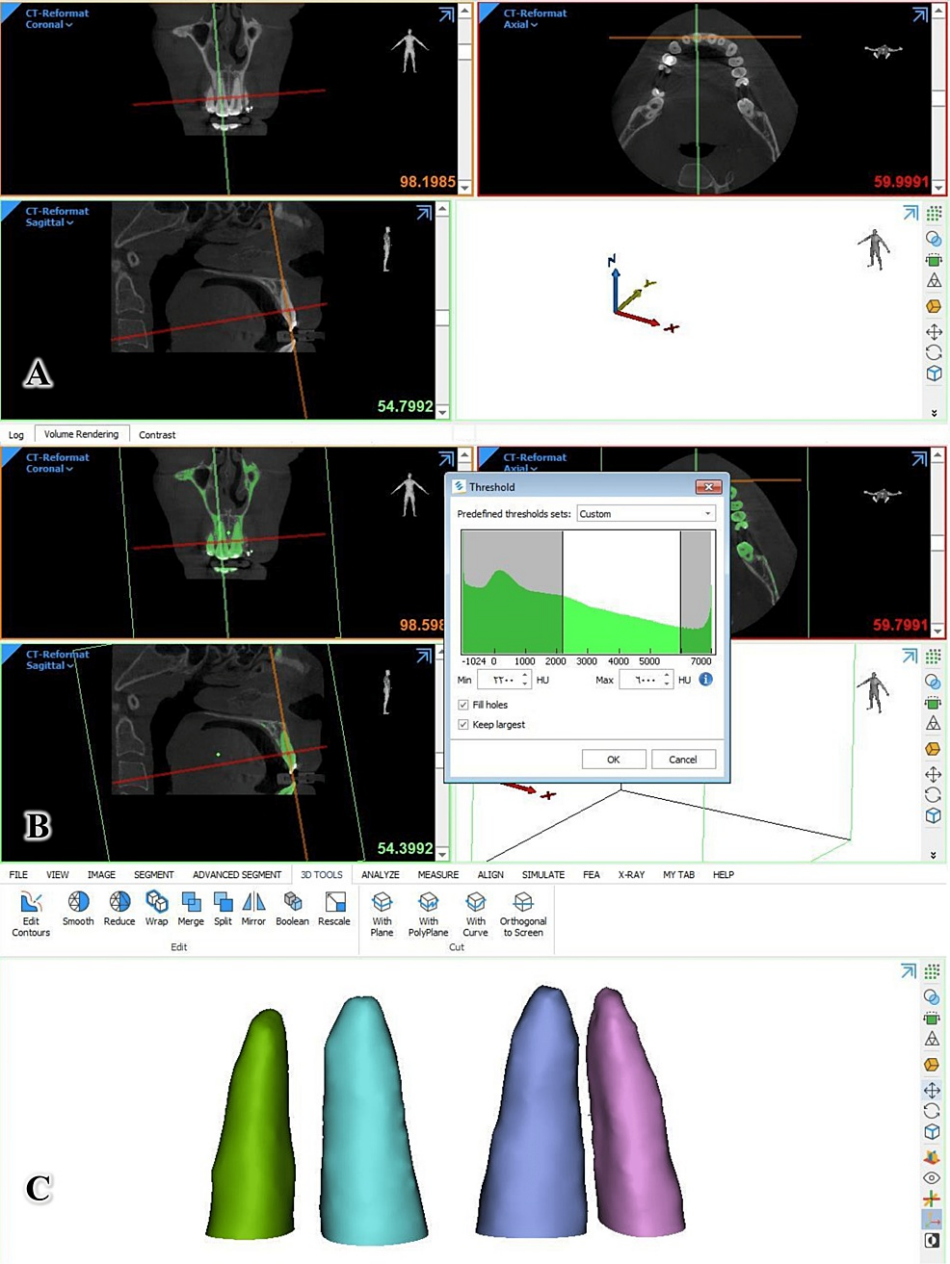
The software used to view and manipulate the DICOM files from CBCT Images. This software is also used to construct the roots of the upper incisor teeth. A: Orientation of the axes in the three dimensions. B: Determination of the appropriate intensity for each patient individually through the "Threshold" window. C: Once the 3D models of the upper incisors' roots were constructed, their volumes could be calculated DICOM: Digital Imaging and Communication in Medicine; CBCT: cone-beam computed tomography.

Mimics Research™ V21.0 (Materialise NV Technologielaan, Leuven, Belgium) was used to view and manipulate the DICOM files. The condyles were directed in the three planes (axial, frontal, and sagittal) according to the longitudinal axis of each incisor. This was accomplished by using the "Along Plane" tool, which can be opened from the "VIEW" window by selecting "Interactive MPR," moving to the "SEGMENT" window, and choosing "New Mask" to determine the density (Figure 5). The appropriate density was chosen for each patient individually [22] so that the lowest possible thickness of the tissues surrounding the upper incisors and the largest possible amount of the dental tissues of these upper incisors appear mechanically. After that, the "Multiple Slice Edit" tool was used to manually remove the tissues surrounding the upper incisors [22] and gradually in layers, with a new mask made in several layers jointly to the dental tissues of the upper incisors only. In the last step, the volume of each root is determined separately by pressing the button "properties." The volumetric root resorption was calculated by subtracting the root volume before and after the intrusion.

Linear Assessment of the Roots

**Figure 6:**
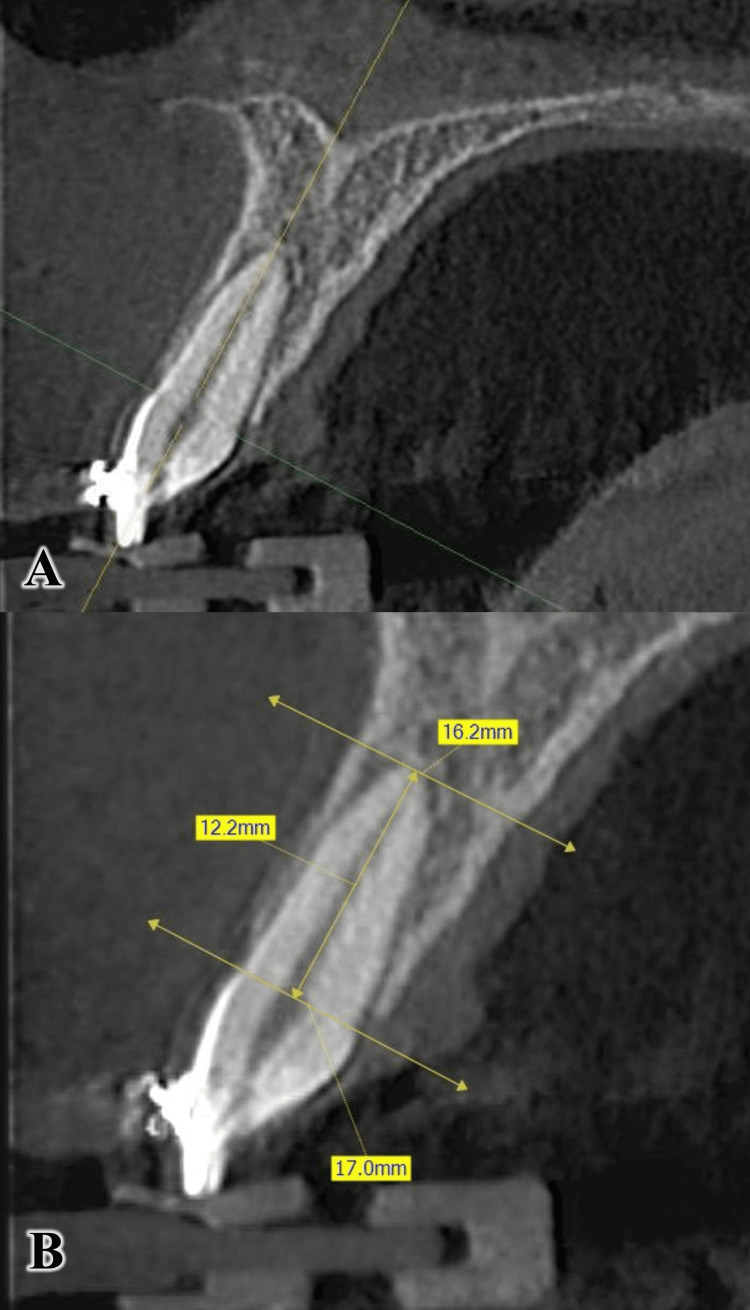
Calculating the root length on CBCT images in the sagittal view. A: Orientation of the two axes (i.e., the frontal and the axial) on the sagittal view. B: Measuring the distance between the two parallel yellow lines to determine the root length CBCT: cone-beam computed tomography.

The line passing through the cementoenamel junction (CEJ) was adopted as a reference line [8,23]. In the sagittal section, the axial and frontal axes were directed in the three levels so that they passed along the longitudinal axis of the tooth, with the sagittal axis perpendicular to the two previous axes. Two lines were drawn, one passing through the CEJ and the other passing from the root apex so that they were perpendicular to the tooth's axis. After measuring the distance between the two lines, the root length was determined (Figure 6). The linear resorption was calculated by subtracting the root length before incisor intrusion from the root length after incisor intrusion.

The error in the method

Twelve CBCT images were chosen at random from all of the images and measured a month following the initial evaluation again. Intraclass correlation coefficients were used to assess reliability, and they demonstrated strong reliability with ranges between 0.925 and 0.999. As measured by the systematic error, there was no statistically significant difference between the two measurements using a paired t-test.

Statistical analysis

Statistical analysis was attained using the SPSS statistics software (version 25.0, IBM, Armonk, NY, USA). The Shapiro-Wilk test was employed to test the normality of data distribution, which revealed normal distribution; hence, parametric tests were applied. A paired t-test was used for intergroup comparisons of the measurements, whereas an independent t-test was applied to evaluate the differences between the two groups. At 0.05, the significance level was determined.

## Results

Baseline sample characteristics

Thirty patients (13 males, 17 females; mean age 22.4±3.37 years) were enrolled and distributed to the laser or the control groups (Table 1). There were no dropouts, and all patients received a complete follow-up. The mean treatment period was 4.64±0.76 months in the laser group and 4.71±0.97 months in the control group.

**Table 1:** Baseline sample characteristics n: number; SD: standard deviation.

Group (n)	Gender (n)		Age in years
	Male	Female	Mean (SD)
Laser (15)	5	10	21 (3.07)
Control (15)	8	7	23.80 (3.16)
Total (30)	13	17	22.40 (3.37)

Changes in root volumes

There was a volumetric decrease in both groups' upper central and lateral incisors roots, which was statistically significant (P<0.001; Table 2). When comparing the two groups, it was found that the amount of decrease occurring in the upper central incisors in the laser exposure group was less than the amount of decrease in the control group, but the difference was not statistically significant (P=0.345; Table 3). The same applies to the amount of decrease in the upper lateral incisors in the laser group. It was less than that seen in the control group, but the difference between the two groups was statistically insignificant (P=0.263; Table 3).

**Table 2:** Descriptive statistics of the root volumes for both the LLLT and the control groups, as well as the P-values of significance testing within each group *Significant difference. U1: central incisor; U2: lateral incisor; SD: standard deviation; LLLT: low-level laser therapy.

Variable	LLLT group					Control group				
	T1		T2		P-value	T1		T2		P-value
	Mean	SD	Mean	SD		Mean	SD	Mean	SD	
U1 (mm³)	268.28	28.29	248.76	31.28	<0.001*	274.91	32.71	252.98	35.66	<0.001*
U2 (mm³)	207.97	33.31	187.54	29.06	<0.001*	214.03	27.82	190.97	25.10	<0.001*

**Table 3:** Descriptive statistics of the change in root volumes for both the LLLT and the control groups, as well as the p-values of significance testing between the two groups T1: before intrusion; T2: following  intrusion; U1: central incisor; U2: lateral incisor; SD: standard deviation; LLLT: low-level laser therapy.

Variable	LLLT group		Control group		P-value
	Mean change (T2-T1)	SD	Mean change (T2-T1)	SD	
U1 (mm³)	-19.52	6.72	-21.93	7.00	0.345
U2 (mm³)	-20.43	6.23	-23.06	6.38	0.263

 Changes in root lengths

There was a linear decrease in upper central and lateral incisors roots in both groups, which was statistically significant (P<0.001; Table 4). When comparing the two groups, it was found that the amount of decrease occurring in the central incisors in the laser exposure group was less than the amount of decrease in the control group, but the difference was not statistically significant (P=0.343; Table 5). The same applies to the amount of decrease in the lateral incisors in the laser exposure group. It was less than that seen in the control group, but the difference between the two groups was not statistically significant (P=0.461; Table 5).

**Table 4:** Descriptive statistics of the root lengths for both the LLLT and the control groups, as well as the P-values of significance testing within each group *Significant difference. U1: central incisor; U2: lateral incisor; SD: standard deviation; LLLT: low-level laser therapy.

Variable	LLLT group					Control group				
	T1		T2		P-value	T1		T2		P-value
	Mean	SD	Mean	SD		Mean	SD	Mean	SD	
U1 (mm)	12.76	1.74	12.05	1.86	<0.001*	13.10	1.31	12.30	1.25	<0.001*
U2 (mm)	12.92	1.46	12.09	1.49	<0.001*	12.52	1.54	11.57	1.51	<0.001*

**Table 5:** Descriptive statistics of the change in root lengths for both the LLLT and the control groups, as well as the P-values of significance testing between the two groups T1: before intrusion; T2: following intrusion; U1: central incisor; U2: lateral incisor; SD: standard deviation; LLLT: low-level laser therapy.

Variable	LLLT group		Control group		P-value
	Mean change (T2-T1)	SD	Mean change (T2-T1)	SD	
U1 (mm)	-0.70	0.31	-0.80	0.23	0.343
U2 (mm)	-0.83	0.40	-0.94	0.44	0.461

Harms

During this study, no harm or unfortunate effects were observed.

## Discussion

This is the first RCT investigating the effectiveness of LLLT on OIIRR of the upper incisors during intrusion movement using mini-implants in adult patients with a deep overbite.

For orthodontists, intrusion movement is a difficult and challenging procedure and is often performed for esthetic and functional reasons [24,25]. On the other hand, OIIRR has been described as an inescapable pathologic consequence of orthodontic tooth movement [7]. In this context, LLLT has been used recently to improve the mechanisms of orthodontic movement, as many studies have shown that an adequate biological response has occurred in the periodontal ligament with the possibility of reducing the risk of root resorption [12]. In previous studies, laser irradiation has been used with varying wavelengths, energies, exposure times, application sessions, and orthodontic movements [14,26-28]. A diode laser with an 808 nm wavelength was used in this study, which was in the optimal wavelength range and penetrated well into the tissues. The used laser had the following parameters: 250 milliWatt power output, 4 Joules/point energy density, and 16 seconds irradiation per point, and was applied to each tooth of the upper incisors. To the best of our knowledge, no previous studies have dealt with the effect of laser on root resorption during intrusion movement using implants in adult patients with a deep overbite.

CBCT was selected in this study because it is the most reliable tool for diagnosing external root resorption compared to two-dimensional radiography methods [8]. In addition, CBCT images could provide three-dimensional (3D) data, enable practitioners to evaluate 3D stereological root resorption, and be used to assess 3D volumetric changes [22,29]. A force of 40 g was chosen for the intrusion at each side (20 g for each incisor) as it is considered the best force for intrusion and causes the least damage to the periodontal tissues, according to Burstone [21]. Compared to traditional orthodontic appliances, mini-implants provide more effective anchorage control for complex orthodontic tooth movements, such as intrusion [24].

The results of the current study showed statistically significant differences in the volumes and lengths of roots before and after the intrusion within the laser and the control groups (P<0.05). At the same time, root resorption occurred on all the maxillary incisors in both groups after the end of the intrusion stage. On the other hand, the volumetric root resorption of the upper central and lateral incisors was reduced by 10.9% and 11.4%, respectively, in the LLLT group compared with the control group. Furthermore, the linear root resorption of the central and lateral upper incisors decreased by 12.5% and 11.7%, respectively, in the LLLT group compared to the control group. However, those differences between the two groups were statistically insignificant for all measurements (P>0.05).

Low-level laser did not significantly reduce the root resorption resulting from dental movements. This result was similar to many previous studies, which have not shown any effect on OIIRR [14,27,28,30].

In contrast, the study of both Ng et al. and Fernandes et al. found that the low-level laser effectively reduced root resorption, and the differences were statistically significant [7,31]. The disagreement with the study of Ng et al. may be attributed to the mean age of their sample, which was 16.4 years for males and 16.7 years for females. Another reason could be the accomplished dental movement, which was a tilt of the first premolars by applying a force of 150 g before their extraction. In addition, the area to which the laser was applied (i.e., the first premolars area) and the laser application protocol in Ng et al.'s study differed from those in the current trial [7]. As for Fernandes et al.'s study, the mean age in their study was higher (i.e., 35-65 years) compared to the current study (i.e., 18-30 years). In addition, the area to which the laser was applied (the maxillary first molars on both sides) was 150 g of intrusion force, and the laser application protocol was different from the one employed in the current study [31].

LLLT is an effective tool in accelerating orthodontic tooth movement, as shown in many previous studies [10-12]. One of the strengths of the current trial is the evaluation of its efficacy in reducing the amount of root resorption regardless of its effect on the speed of orthodontic movements. Unfortunately, many previous reports evaluating the effectiveness of accelerated orthodontics have not considered the evaluation of root integrity when surgical, physical (e.g., LLLT), and biomechanical methods have been used [32-36]. Although some acceleration-based trials have evaluated root morphology following orthodontic tooth movement, their analysis has only been confined to 2D assessment [37].

Limitations

The main limitations of the current study are no long-term follow-up period for root resorption, the limited distance between the roots of the upper central and lateral incisors to insert the mini-implants, although it was within the distance of safe placement of mini-implants, and the esthetic view of springs and mini-implants in the anterior region. In addition, the present study does not address genetic differences in the tendency to resorption.

## Conclusions

When mini-implant-based incisor intrusion was accomplished, a small amount of root resorption was found in both groups. However, this resorption did not exceed 16% of the upper incisor's total root volume or length following intrusion. The low-level laser irradiation using the current protocol did not significantly affect the amount of root resorption induced by incisor intrusion in the experimental group compared to the control group.
